# Case of Wound of the Brachial Artery

**Published:** 1827-06

**Authors:** 

**Affiliations:** *the* Westminster Hospital


### WOUNDS OF ARTERIES.

### *Case of Wound of the Brachial Artery, treated at the* Westminster Hospital, *by* Mr. White.

Mr. White was requested to see Thomas Taylor, who was a groom at Astley's Amphitheatre, and who had been bled by a druggist a few days previously. He was visited by Mr. White at the request of Mr. Ridge, an apothecary; and it was stated that the phlebotomist had accidentally wounded an artery. Frequent hemorrhage during the three following days, with considerable enlargement of the arm, sufficiently warranted the supposition. Mr. White requested that he should be forthwith sent to the hospital, which was not done for two days.

On August 4th the man was received, and on the same night considerable hemorrhage took place, which was restrained by compressing the subclavian artery until the arrival of Mr. White. The arm was now prodigiously swollen, both above and below the flexure of the joint, lividly red, and extremely painful on the slightest pressure, peremptorily forbidding any attempt at compression with pad and bandage. The orifice was plugged with a coagulum, which for a time appeared to restrain the bleeding.

Mr. White, from the previous history of the case and the existing condition of the arm, and also taking into consideration the great loss of blood which had occurred at intervals daily since the accident, stated his opinion that it was not only not safe to leave the patient to the chance of more hemorrhage during the night, but that the tense and exquisitely sensitive condition of arm, which he conjectured was gorged with coagulated blood, demanded an extensive division of the integuments, to procure immediate relief. A curved director was passed into the orifice, and conveyed under the integuments nearly its whole length upwards, and afterwards equally low downwards, making a free opening of eight inches with a curved bistoury. The subclavian artery was at the time compressed. Immediately on the division of the skin, several ounces of coagulum were let free, and rolled out without assistance; the rest was removed with a sponge and water: there appeared an accumulation of at least two pounds of coagulum. Pressure being removed from the subclavian artery, an instantaneous gush of blood immediately directed the operator to the wounded vessel, which was found, after a little separation of the surrounding parts, to be distinctly the brachial artery. A ligature was placed above the wounded point of the vessel, and, to prevent hemorrhage by regurgitation from the vessels of the forearm, another was placed below. The two ligatures, with the intermediate artery, included an inch of the vessel. The test of the security of the wounded vessel was the cessation of all hemorrhage when pressure was removed from the subclavian. The highly inflamed state and tension of the arm forbade any attempt at union by suture. A small quantity of dry lint was lightly, therefore, placed in the wound, and a soft bread poultice over the whole arm; which was afterwards supported on a pillow.

August 5th.—Pain in the arm much diminished; pulse 120, and feeble.

Six P.M.—Increase of arterial action. This continued till about four o'clock in the morning of the 9th, when the artery burst out bleeding: this was restrained by pressure applied on the subclavian artery. About nine A.M. Mr. White again put a ligature on the brachial artery, a little above the bleeding point. A dose of laudanum was given in some brandy, which was rejected by vomiting. Pulse in the opposite arm very feeble, but rose in a few hours. The patient now got some sleep. At four P.M. Mr. White saw him: pulse 120. Ordered a dose of cathartic medicine.

7th.—Has had some sleep during the night, and says he feels a great deal better. Pulse 110, and hard.

8th.—Has had but little sleep; complains of increase of pain in the arm; pulse 104; bowels open. The arm has returned to its natural colour, but is still very hard about the biceps muscle.

9th.—Has passed a restless night; great pain in the arm; bowels open, though the tongue is very much furred; pulse ninety-six, hard and full.

10th.—Has had a little sleep; pain continues; rather feverish.

Ordered Mist. Salin. ℥ viij.; Træ. Opii gtt. l. Capiat cochl. iij. ter die.

11th.—Has passed a good night; arm less painful; bowels have been freely opened; pulse ninety-six.

August 12th.—Has had a good night's rest; very little pain in the arm; wound beginning to slough; pulse 100; bowels not open.

Ordered Ext. Coloc. C. gr. viij.

13th.—The nurse, on removing the dressing this morning, discovered a large quantity of blood. Mr. White was immediately sent for. He found that the lower extremity of the vessel was the point from which the hemorrhage issued, and a ligature was placed upon it.

14th.—He feels a great deal of pain and heat in the arm.

Ordered Ext. Opii gr. j.

15th.—Patient feels better; has passed a quiet night; pulse weak and feeble.

16th.—Great pain in the arm, and throbbing about the wound.

Capiat Pulv. Ipecac. comp. gr. viij.

17th.—Much as yesterday. Pulse 104.

18th.—At four this afternoon, a slight hemorrhage came on, to the extent of about four ounces: the blood was venous, and ceased spontaneously. Pulse soon after rose from 96 to 120.

Eight P.M.—Pain still continues; pulse 110.

19th.—Feels himself a little better.

20th.—Continues better. Bowels confined.

Ext. Coloc. C. gr. viij.

21st.—Not so well. Bowels confined; pulse hard and quick; tongue furred.

Ordered Hydrarg. Submur. et Ext. Coloc. C. āā gr. v. fiant pil. ij. statim sumendæ.

22d.—Better in every respect; had a good night's rest; bowels freely opened; pulse full.

About eight P.M. the arm again burst out bleeding, when he lost thirty ounces. Hemorrhage restrained by pressure on the brachial artery. Mr. White being out of town, Mr. Wm. Lynn placed a ligature an inch above the bleeding orifice. Ordered Conf. Opiat. ℈j. Half an hour after this, a slight hemorrhage recurred, when Mr. Guthrie saw him; but the bleeding ceased on the application of a compress.

23d.—Has had no sleep last night. The patient throughout has had nourishing diet, with wine and brandy.

24th.—This morning about sixteen ounces of blood were lost. Mr. White instantly proposed amputation to the patient, as the only probable chance of saving him from another bleeding, which would most likely prove fatal. The patient consenting, the operation was immediately performed, during which but little blood was lost.

Ordered Træ. Opii gtt. l. in a little Cinnamon water.

Four P.M.—Feels very restless, not at all inclined to sleep.

25th.—Better. Pulse full and soft; has passed a good night; complains of but little pain in the stump; bowels rather relaxed; skin cool and moist.

Four P.M.—Has been purged five or six times since ten o'clock.

Ordered Pulv. Cinnam. comp. gr. xv. omni horâ.

August 26th.—Says he is better. Has had a good night's rest. Pulse full and soft; bowels regular; skin cool and moist; but little pain in the stump.

Eight P.M.—Diarrhœa returning.

Ordered Pulv. Rhei gr. x. in Aquæ Menth. Pip.

27th.—Has had a discharge of dark green coloured stools. Violent increase of the heart's action.

Ordered Hydrarg. Submur. gr. ij. et Ext. Opii gr. ss. sexta quâque horâ.

28th.—Very much debilitated. Dressings removed from the stump: there is some union between the lips of the wound.

Four P.M.—Diarrhœa continues.

Ordered Mist. Cretæ ℥vss.; Confect. Opiat. ℈ij. M. capiat cochl. ij. quartâ quâque horâ.

Eleven P.M.—Ol. Ricini ʒij.; Mucil. Acaciæ q. s.; Aquæ Menthæ ℥jss.; Træ. Opii gtt. x. fiat haust. statim sumend.

29th.—Has had four very scanty evacuations during the night. Stump dressed to-day: has a favourable appearance.

From this period the patient's health and strength rapidly declined till he died.

This very interesting case having at the time excited considerable attention, and various contradictory statements having been published respecting many of the circumstances, we have procured notes of it from a gentleman who witnessed its progress, and sent them to Mr. White, requesting his permission to publish them if correct; and at the same time expressing our wish that he would favour us with any comment or observations on the subject. We are indebted to his politeness for the following reply :—

### Mr. White's *Observations.*

The observations which I had an opportunity of making during the progress of Taylor's case are as follow :

The accident having occurred four or five days before he was received as my patient into the hospital, the man's mind had become excessively alarmed at the frequent recurrence of hemorrhage, added to the anxiety which he evidently saw expressed by those around him, and thus creating a very unfavourable impression on his imagination,—viz. that the accident, of which he had been made aware, was likely to terminate unfavourably. This unceasing mental excitement, with great loss of blood and considerable local pain, had rendered him unfit to undergo any surgical operation. The pulse was hurried and irregular, his tongue coated, the stomach irritable, with thirst and throbbing headache; and he had passed three or four sleepless nights. The local urgency presented by the state of the arm, combined with the danger of a postponed examination as to the return of hemorrhage, determined me, at twelve o'clock at night, to proceed in the manner above detailed.

On the following day, although the patient's countenance was less expressive of anxiety, yet it was far from having that quietude which I should have been gratified to have met. He had slept at intervals, and the arm was less painful. The peculiar character of the pulse, with which every practical surgeon is acquainted, and which he denominates *hemorrhagic*, was too evident at this early time; but it existed before the artery was tied, (I had almost said secured.) A throbbing heart, a pale face, with a peculiar acute angular expression, was never lost; and on the fourth day I was called in consequence of fresh bleeding from the wound. It had been restrained by the house surgeon; yet, not trusting to the now very sloughy state of the wound, I proceeded to remove the coagulated blood which filled the capacious cavity, and I found both the original ligatures firm in their places. After the repeated application of a warm sponge, this hemorrhage appeared to proceed from a point immediately below the upper ligature: I therefore passed directly under that point another, which put a stop to further bleeding.

Five or six days now passed away, the condition of the patient fluctuating as to general health, but no "laudable" suppuration was secreted in the wound; and although, on the whole, I considered him in a better state, yet he still was in a very critical situation.

When I did not expect it, (the patient being, as I conceived, somewhat improved in general health,) hemorrhage again took place; and, after a most minute examination, for it was, in the deep and sloughing wound, extremely difficult to ascertain the precise point where to dip the needle or elevate with the tenaculum, I succeeded in restraining further hemorrhage, by including with a ligature the vessel and a considerable portion of the surrounding spongy structure.

Early the next morning, I was professionally called away out of town. Hemorrhage again occurred; and Mr. William Lynn placed a ligature on the brachial artery, above the point originally tied.

The next day, Sir A. Carlisle and Mr. Guthrie saw the patient, and, from the exceedingly exhausted state of the man, they conceived that, independent of the improbability of a stump after amputation, under such circumstances, doing well, the exhausted state of the patient forbade that operation.

I was called to the man at noon two days subsequently, for a further hemorrhage had at that time happened, and fourteen or sixteen ounces had been lost before it was discovered. On my arrival, thinking that even in this worn-out condition of the patient, whose courage now and throughout had been the most unshaken, and although, from a previous consultation, he had been fully persuaded that his case was to be speedily fatal, yet, on being now told that the removal of the arm still offered him a chance of salvation, he cheerfully consented, and suffered the operation without an expression of impatience or pain.

The examination of the arm after amputation clearly showed that the brachial artery (not the radial or ulnar, as has been erroneously stated,) had been wounded; and that the sloughing of the structures at the bottom of the wound had extended to the commencement of the radial and ulnar arteries, thus permitting a regurgitating flow of blood. Notwithstanding the enormous quantity of blood lost at intervals, and the frequent necessity of disturbing the parts, which could not be done without creating great pain, and much exhaustion had been produced, I yet felt sanguine of the man's recovery. Diarrhœa, however, after a few days impossible to control, was superadded; and, although the stump was partially healed, and the ligatures remained firmly on the arteries, he gradually sunk, and died.

If the same accident were again to happen, and *under the same circumstances,* I am not aware that any preferable mode of practice could be adopted, if it is contemplated to save the limb. The failure of the case is entirely attributable to the repeated sloughing of the wound and arteries; a circumstance not unfrequent after ordinary amputation, and even after the lesser operations, where a peculiar state of the system has been produced by constitutional disturbance.

